# Large-Scale Mapping of Axonal Arbors Using High-Density Microelectrode Arrays

**DOI:** 10.3389/fncel.2019.00404

**Published:** 2019-09-06

**Authors:** Torsten Bullmann, Milos Radivojevic, Stefan T. Huber, Kosmas Deligkaris, Andreas Hierlemann, Urs Frey

**Affiliations:** ^1^RIKEN Quantitative Biology Center, RIKEN, Kobe, Japan; ^2^Graduate School of Informatics, Kyoto University, Kyoto, Japan; ^3^Carl Ludwig Institute for Physiology, University of Leipzig, Leipzig, Germany; ^4^Department of Biosystems Science and Engineering, ETH Zurich, Basel, Switzerland; ^5^Graduate School of Frontier Biosciences, Osaka University, Osaka, Japan; ^6^MaxWell Biosystems AG, Basel, Switzerland

**Keywords:** axons, high-density microelectrode array, extracellular electrical field, action potential, axonal arborizations, action potential propagation, high-throughput screening

## Abstract

Understanding the role of axons in neuronal information processing is a fundamental task in neuroscience. Over the last years, sophisticated patch-clamp investigations have provided unexpected and exciting data on axonal phenomena and functioning, but there is still a need for methods to investigate full axonal arbors at sufficient throughput. Here, we present a new method for the simultaneous mapping of the axonal arbors of a large number of individual neurons, which relies on their extracellular signals that have been recorded with high-density microelectrode arrays (HD-MEAs). The segmentation of axons was performed based on the local correlation of extracellular signals. Comparison of the results with both, ground truth and receiver operator characteristics, shows that the new segmentation method outperforms previously used methods. Using a standard HD-MEA, we mapped the axonal arbors of 68 neurons in <6 h. The fully automated method can be extended to new generations of HD-MEAs with larger data output and is estimated to provide data of axonal arbors of thousands of neurons within recording sessions of a few hours.

## Introduction

The classical view of axons is that of mere transmission cables (Hodgkin and Huxley, 1952), while dendrites integrate distinct synaptic inputs (Spruston, 2008), and learning and memory are perceived to be a consequence of synaptic plasticity (Redondo and Morris, 2011). Recent data, however, indicate that the functional capacity of axons may be much more complex (Debanne, 2004; Ohura and Kamiya, 2016; Rama et al., 2018).

According to the classical theory describing giant squid axons (Hodgkin and Huxley, 1952), voltage-gated sodium and potassium channels support self-sustained action potentials that propagate along the axon. That is also true for mammalian axons, but at least two additional types of cationic channels have been described. These channels are activated by G-protein dependent receptors or hyperpolarization and modify the shape and propagation of the action potentials (Elgueta et al., 2015; Ko et al., 2016).

Axons can be surrounded by a myelin sheet that leads to saltatory conduction of action potentials (Tasaki, 1939), instead of the continuous conduction that has been observed in non-myelinated axons, such as the giant squid axons (Hodgkin and Huxley, 1952). Mammalian axons also show considerable variations in their diameter (Gaussian distribution around a peak at 200 nm diameter) and their number of branch points and varicosities, which affect the conduction velocity of action potentials. Differences in axon lengths have been found to produce defined temporal delays for spike trains for coincidence detection in the auditory system (Seidl et al., 2010; Stange-Marten et al., 2017). Furthermore, axonal delays in the neocortex show sub-millisecond precision (Swadlow, 1994), which is compatible with mechanisms of spike-timing-dependent synaptic plasticity (Dan and Poo, 2004). These characteristics lead, according to computational studies (Izhikevich, 2006), to the emergence of precisely timed firing patterns.

Early computational studies have suggested that some axonal arbors can act as filters for spike patterns by the selective failure of action potential propagation (Lüscher and Shiner, 1990). Whereas cerebellar mossy fibers in the cerebellum can reliably transmit spike trains up to 1.6 kHz (Ritzau-Jost et al., 2014), occasional failures have been observed in the axons of CA3 pyramidal neuron at firing frequencies of 30–40 Hz (Meeks and Mennerick, 2007). Failure depends on the diameter and the branching morphology as well as on the activation of presynaptic A-type potassium channels. The morphology and molecular composition of the individual axonal arbors has been found to influence the timing and shape of the action potential (Bischofberger et al., 2002; Alle and Geiger, 2006; Cho et al., 2017) arriving at the pre-synapse, which affects synaptic transmission and plasticity. For example, broadening of action potentials due to slow inactivation of voltage-gated potassium channels during high-frequency spike trains was found to facilitate synaptic release (Geiger and Jonas, 2000). Furthermore, axonal signaling and synaptic connectivity have been found to constitute important parameters in induced-pluripotent-stem-cell (iPSC) models of Parkinson's (Kouroupi et al., 2017) and amyotrophic lateral sclerosis (Wainger et al., 2014).

The understanding of information processing in axons is a fundamental question in neuroscience; however, the availability of experimental data is severely limited due to the small axon diameter. Most data sets have been gathered by performing patch clamp recordings of axonal membranes, at axon terminals and boutons, at the intact axon shaft or at the axonal bleb that is formed upon cutting axons (Ohura and Kamiya, 2016). However, these patch-based methods are not very well-suited to track the propagation of action potentials at more than two sites or even across the full axonal arbor. Moreover, the patch recording time is limited to a few hours while axonal recording is a serial process and has to be done axon after axon. Finally, patch-based methods constitute endpoint measurements and following the development of axonal arbors over extended periods is not possible. Another possibility to study axons includes the use of imaging tools and optogenetics (Chen et al., 2013), and it has been shown that imaging of single axon terminals is possible (Hoppa et al., 2014). However these methods are still limited in signal-to-noise ratio as well as in temporal resolution (Emmenegger et al., 2019).

A viable alternative to the methods described above includes the use of high-density microelectrode arrays (HD-MEAs), based on complementary-metal-oxide-semiconductor (CMOS) technology (Eversmann et al., 2003; Berdondini et al., 2009; Frey et al., 2010; Ballini et al., 2014; Bertotti et al., 2014; Viswam et al., 2016; Tsai et al., 2017). HD-MEAs can be used to capture neuronal activity at high temporal resolution across spatial scales, including networks, dendrites and most importantly, axons (Obien et al., 2015). CMOS-based HD-MEAs have been used to study the action potential propagation along axonal arbors (Bakkum et al., 2013) and the initiation of action potentials at the axon initial segment (Bakkum et al., 2019), as well as to track single action potentials along axons (Radivojevic et al., 2017) and stimulate single axon initial segments (Ronchi et al., 2019). Furthermore, HD-MEA recordings can be combined with classical patch clamp recording to study postsynaptic currents in response to pre-synaptic stimulation (Jäckel et al., 2017).

The objective of this work was to provide a method for high-throughput scanning of axonal arbors and mapping their axonal delays with minimal or no need to adjust parameters for the detection of axonal signals. Using a standard HD-MEA (Frey et al., 2010), we mapped the axonal arbors of more than 68 neurons in <6 h.

## Materials and Methods

### Animal Use

Timed pregnant rats (Wistar) were obtained from a commercial vendor (Nihon SLC, Japan). Animals were sacrificed on the day of arrival to obtain embryos for primary neuron cultures. All experimental procedures on animals were carried out in accordance with the European Council Directive of 22 of September 2010 (2010/63/EU) and have been approved by the local authorities in Japan (Animal Care and Use Committee of RIKEN; QAH24-01).

### High-Density Microelectrode Array (HD-MEA)

Sub-cellular resolution extracellular recordings were obtained using a CMOS-based HD-MEA (Frey et al., 2010) with 11,011 electrodes, arranged in a hexagonal pattern and featuring an electrode density of 3,150 electrodes/mm^2^. The culture chamber of the HD-MEA was prepared as described before (Heer et al., 2007) with minor modifications: after attaching the chamber ring (polycarbonate, 19 mm inner diameter, 8 mm high) using epoxy resin (EPO-TEK 301-2, Epoxy Technology Inc.), GlobTop (G8345D-37, Namics Inc.) was used to cover the bond wires while keeping the electrode area clean, and the remaining area was covered by a thin film of PDMS (Sylgard 184, Dow Corning). Platinum black was electrochemically deposited (Marrese, 1987) [with modifications of the original procedure (Heer et al., 2007)] on the electrodes to decrease their impedance in order to improve signal-to-noise characteristics (Viswam et al., 2014). Before plating, the surface of the HD-MEAs was rendered hydrophilic by oxygen-plasma treatment (40 s, 20 W), incubated for 4 h with of 50 μg/ml Poly-D-Lysine (Sigma-Aldrich, P7280) in PBS, washed twice with aqua dest and air-dried for 1 h.

### Primary Neuron Cultures

Adult rats were anesthetized with isofluorane and killed using a guillotine. The embryos were removed from the uterus and decapitated. Their neocortex was dissected in ice-cold dissection medium (HBSS without Ca^2+^ and Mg^2+^; Gibco, NO.14175) and incubated for 20 min at 37°C in Trypsin/EDTA (Sigma-Aldrich). After washing twice with plating medium (Neurobasal A, supplemented with 10% Fetal bovine serum, 2% B27 Supplement, 1:100 GlutaMax, all from Gibco, Japan, and 10 μg/ml Gentamicin, Sigma-Aldrich, Japan), the tissue was mechanically dissociated, passed through a 40 μm nylon mesh, and centrifuged 6 min at 200 g. The supernatant was removed; the cells were suspended and counted. A 20 μl drop containing 10,000 cells was placed on the electrode area of the HD-MEA in the middle of the culture chamber. The cultures were covered with a membrane, permeable to gas but not to water vapor, and placed in a standard incubator (37°C, 5% CO_2_, 80% relative humidity). The neurons were allowed to settle and attach to the surface during 30 min. Thereafter, the culture chamber was filled with 600 μl serum-free, astrocyte-conditioned DMEM/Hams's F12 medium (Nerve Culture Medium, Sumitomo, Japan, #MB-X9501). Medium was completely exchanged with 600 μl conditioned medium after 4 days and then every 7 days until day 17 *in vitro*.

### Recordings and Spike Event Detection

For recording, HD-MEAs were placed in a bench-top incubator (TOKAI HIT, Japan, INU-OTOR-RE) with temperature control, and 5% CO_2_ was supplied by a gas-mixer and humidified by a water bath. In order to avoid the evaporation of medium during prolonged recording intervals, the water bath and the lid temperature set point was set 1K and 3K above the sample temperature set point, which was 35°C. The HD-MEA recordings were performed using custom scripts, written in LabView (National Instruments, US), Matlab (Mathworks, US), C++ and Python running on a standard PC with a Linux operating system. Data underwent lossless data compression and were directly stored on a server on the local LAN.

**Figure 1:**
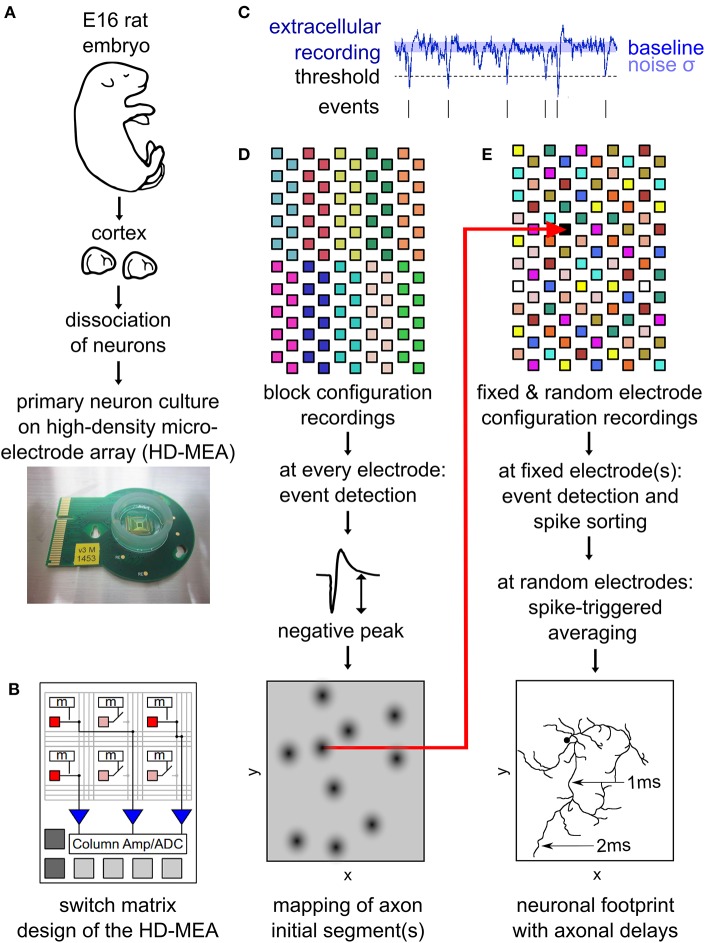
Outline of the main experimental methods. Neurons from embryonic-day-16 rat cortex were cultured on high-density microelectrode arrays (HD-MEAs) **(A)**. The HD-MEA has a high number and density of electrodes in the recording area and a limited number of low-noise on-chip amplifiers placed at the periphery. A switch matrix allows for using different recording configurations by selecting recording electrodes and connecting them to the amplifiers **(B)**. Extracellular recordings enabled the detection of spiking events by a simple thresholding procedure **(C)**. The amplitude of the negative peak of these spikes was mapped across the entire HD-MEA by recording from all electrodes using non-overlapping block configurations **(D)**. This map reveals the location of individual neurons, as neuronal axon initial segments (AISs) are strong contributors to a neuron's local extracellular field potential. Neuronal footprints (see, e.g., Figure 2B) were obtained by recording with multiple configurations consisting of both, fixed electrodes, as well as randomly selected electrodes **(E)**. The fixed electrodes (indicated in black) were located near the AIS of the neuron and did not change between the configurations. The remaining electrodes of the HDMEA where sequentially recorded in batches (indicated by the same color) consisting of electrodes at randomly selected locations. Spike sorting of the spike events on the fixed electrodes was performed to obtain the activity of single neurons. Spike-triggered averages from all configurations were then combined into a single footprint for each neuron revealing the axonal delays **(E)**. **(B)** was adapted with permission from Obien et al. (2015).

Offline analysis of the recordings included filtering, event detection and averaging. First, a band-pass filter (2nd order Butterworth filter, 100–3,500 Hz) was used to remove slowly changing field potentials as well as high frequency noise. The remaining (background) noise was characterized by the median absolute deviation (MAD), which is resilient to outliers in the data but represents a consistent estimator of the standard deviation, *s*_*V*_ = 1.4826 *MAD*(*V*_*sig*_). Using a voltage-threshold method for event detection (Lewicki, 1998), negative signal peaks below a threshold of *V*_*thr*_ = 5*s*_*V*_ (V_thr_ > 50 μV in all cases) were identified (Figure 1C). To avoid multiple detection of the same spike, successive events within <0.5 ms were discarded.

### Mapping of Axonal Initial Segments

**Figure 2:**
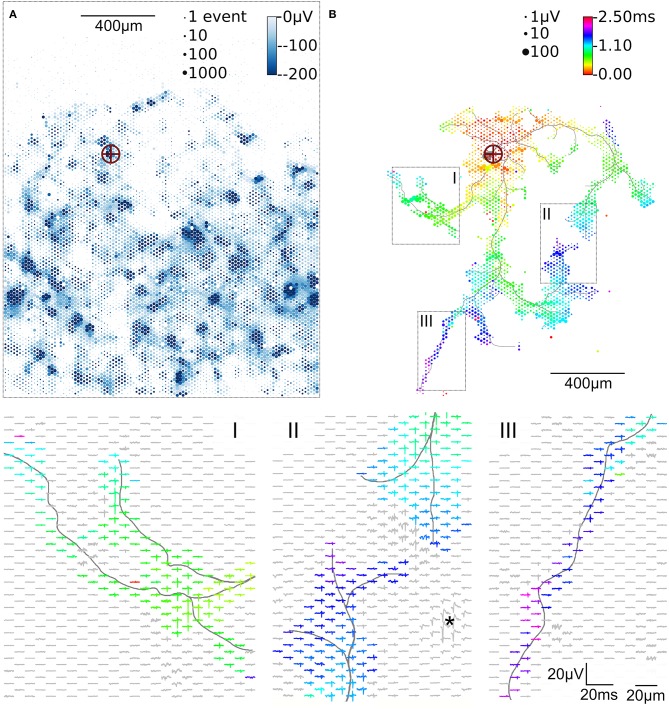
Activity map and footprint of an example neuron. The marker in the activity map reveals the location of the AIS of the example neuron **(A)**. The circle size indicates the square-root-scaled count of spiking events per electrode. The median negative amplitude of the spikes is color-coded with a cut-off at −200 μV. Spike triggered averaging shows the axonal footprint **(B)**. The circle diameter indicates the square-root-scaled amplitudes of the average APs. The axonal delay is color-coded. Close-ups of 3 regions (labeled I, II, III), showing the average AP waveforms, are presented in the lower panels. Gray axonal contours serve as guide to the eye and are estimated by observing the spatial movement of signal peaks in consecutive movie frames (Radivojevic et al., 2017). The asterisc (^*^) indicates an artifact probably from a large spike at the AIS of a presynaptic neuron.

To initially identify the location of axonal initial segments (AISs), the whole array was scanned using configurations in which non-overlapping blocks of 6 × 17 electrodes (Figure 1D) were connected to the amplifiers through the switch matrix (Frey et al., 2010) (schematic in Figure 1B). After recording for 30 s from every electrode in the array, spike detection was performed for each electrode and taking the median value summarized the amplitude of the negative peaks. The median of the negative peak for each electrode was plotted as a map showing some areas with large negative peaks (Figure 2A). Such local minima were assumed to indicate the putative (proximal) AIS locations (Bakkum et al., 2019). We did not distinguish between inhibitory and excitatory neurons according to the AIS waveform (Mita et al., 2019).

### Mapping of Electrical “Footprints”

To map the electrical “footprint” (spatial distribution of extracellularly measured electrical potentials obtained with the densely packed electrodes) of neuronal units and their axonal arbors over the entire array, we used a series of configurations in which so called “fixed electrodes” were always connected to the amplifiers through the switch matrix (Frey et al., 2010), whereas the remaining “variable electrodes” were connected in sequential configurations (schematic in Figure 1E). After recording the spontaneous activity for 115 s for each of these configurations, spike sorting was performed and the electrical “footprint” was calculated using the spike-triggered average as previously described Bakkum et al., 2013; Müller et al., 2015; Deligkaris et al., 2016; Radivojevic et al., 2016, 2017;Jäckel et al., 2017.

To perform spike-triggered averaging we need to reliably record spiking activity of the neurons. Therefore, we selected the fixed electrodes at the putative locations of the AISs but imposed a spatial restriction in order to not record the same neuron twice. For selection of fixed electrodes, all electrodes were ranked according to their median negative peak amplitude (see previous section). The electrode with the highest rank was selected, afterwards all electrodes in its proximity (within 100 μm distance) were discarded from the list, and the procedure was repeated.

### Spike Sorting

Even in sparse cultures some electrodes will pick up spikes from multiple neurons in their vicinity. Therefore, spike sorting was performed for each fixed electrode after recording. Waveforms were extracted for a period of 1.5 ms before to 1.5 ms after the negative peak of each event comprising 61 samples for each event. In order to extract those features that best separate the different clusters of spikes, we performed a principal component analysis. We choose the first 10 principal components as spike features (Abeles and Goldstein, 1977), containing more than 85% of the energy of the signal. KlustaKwik (Kadir et al., 2014) was used to fit a mixture of Gaussians with unconstrained covariance matrices and automatically selected the number of mixture components. Clusters containing more than 2,500 spiking events in total were assigned to distinct neurons. This number corresponds to an average of 14 spikes in each of the 179 recording configurations. With 14 samples the spike triggered averaging reduces the noise by approximately a factor $\frac{1}{\sqrt{14}}=0.27$. This procedure omits neurons with a firing rate below 14 spikes/115 s ≈ 7 spikes/min for which we could not compute a reliable footprint. The uniqueness of the detected neurons was confirmed by assessing their different footprints (**Figure 6**). Clusters leading to identical footprints were manually merged to single neurons, and the footprints of the merged neurons were re-calculated.

### Optimal Recording Configurations for High-Throughput Scanning of Electrical Footprints

The extracellular signals originating from axons and dendrites are very small with respect to the background electrical activity and noise, so that spike-triggered averaging was applied. We developed a set of recording configurations to map the electrical footprint of several neurons in parallel by utilizing the switch matrix of our HD-MEA. The switch matrix can be dynamically configured to connect a large number, *e*, of electrodes to a smaller number, *a*, of amplifiers. In a first-order approach, one could use one electrode as trigger and the remaining electrodes to scan the neuronal footprint, which would result in a large number of configurations, *c*_*w*_, needed to scan the whole array, *c*_*w*_ = *e*/*a*. For recording axonal arbors of *n* neurons, electrodes near the AISs of these *n* neurons have to be always connected to amplifiers (*n* fixed electrodes). The remaining amplifiers can then be connected to the remaining electrodes in several successive configurations (variable electrodes). Following this procedure, the whole array can be scanned with

$$\begin{array}{l} c\left(n\right)=\frac{e-n}{a-n} \end{array}$$

configurations. For *n* neurons we need *c*(*n*) configurations, which means on average *C*(*n*) = *c*(*n*)/*n* configurations for one neuron. An optimal strategy means to choose n in such a way that *C*(*n*) → *min* for 0 < *n* < *a*. With *n* < *a* ≪ *e*, the number of neurons being much smaller than the number of electrodes, we can approximate:

$$\begin{array}{l} C\left(n\right)=\frac{e-n}{\left(a-n\right)n}\approx \frac{e}{\left(a-n\;\right)n} \end{array}$$

The right-hand side has a minimum for *n* = *a*/2. Therefore, approximately half of the amplifiers should be connected to fixed electrodes. The other half of the amplifiers can then be used to scan the whole array in 2*c*_*w*_ configurations, or on average $C_{a}=C\left(a/2\right)=4e/a^{2}$ configurations per neuron. The axonal arbors of a single neuron may extend over the whole array, but by scanning the axonal arbors of many neurons in parallel, the average number of configurations, *C*_*a*_, per neuron is much less than the number of configurations, *c*_*w*_, required for scanning the whole array for large *a*:

$$\begin{array}{l} C_{a}=\frac{4e}{a^{2}}\ll \frac{e}{a}=c_{w} \end{array}$$

This is due to the fact that increasing the number of amplifiers quadratically decreases the average time to scan a single neuron, which allows for high-throughput acquisition of axonal delay maps.

### Live Imaging

Live-cell visualization of whole neurons was performed by transfection (Bakkum et al., 2013; Radivojevic et al., 2016). Transfection was performed using a pLV-hSyn-RFP plasmid from Edward Callaway (The Salk Institute, US; Addgene, #22909) and Lipofectamine 2000 (Life Technologies) in accordance with the manufacturer's protocol. A Leica DM6000 FS microscope, a Leica DFC 345 FX camera, and the Leica Application Suite software were used to produce the micrographs.

## Results

To initially identify the location of axonal initial segments (AISs), the whole array was scanned by using configurations in which non-overlapping blocks (Figure 1D). The median of the negative peak for each electrode was plotted as a map showing some areas with large negative peaks (Figure 2A). Such local minima were assumed to indicate the putative (proximal) AIS locations (Bakkum et al., 2019). In low-density cultures, several AISs could be distinguished (Figure 1A). So called “fixed electrodes” were then selected as trigger electrodes at the putative locations of the AISs, while the remaining “variable electrodes” were selected in sequential configurations to map the electrical footprint of neuronal units and their axonal arbors (Bakkum et al., 2013; Müller et al., 2015; Deligkaris et al., 2016; Radivojevic et al., 2016, 2017; Jäckel et al., 2017) over the entire array (Figure 1E). After spike-triggered averaging, the amplitude and delay of axonal signals could be mapped across the whole recording area for each individual neuron (Figure 2B).

### High-Throughput Scanning of Electrical “Footprints”

We used HD-MEA with 11,011 electrodes and 126 amplifiers (Frey et al., 2010). We connected 62 amplifiers to fixed electrodes and used the remaining amplifiers to scan the whole array selecting 64 electrodes at random positions, which would yield a total of $c\left(n\right)=\frac{e-n}{a-n}=\frac{11011-62}{126-62}=\frac{10949}{64}=172$ configurations (see section Spike Sorting). However, due to the design of the switch matrix in our HD-MEA, some electrodes cannot be selected in the same configuration, which increased the number of recording configurations to 175 (see Table 1).

**Table 1:** Comparison of high-throughput mapping of axonal delays using different types of CMOS-based HD-MEA.

		**This work**	**Extrapolated performance using other HD-MEA**			
**HD-MEA**		**(Frey et al., 2010)**	**(Ballini et al., 2014)**	**(Dragas et al., 2017)**	**(Yuan et al.**, **2018****)**	
Mode		SM	SM	SM	SM	APS
Electrodes		11,011	26,400	59,760	8,640	8,640
Amplifiers		126	1,024	2,048	112	9,216
Configurations		179	51	57	153	1^d^
Neurons^a^		**62**	**512**	**1,024**	**56**	**1,000**^e^
Configurations/neuron		2.8	0.1	0.1	2.7	0.001
Circuit noise (AP band)	μV_RMS_	2,4	2,4	2,4	2,3	12,4
Total noise (AP band)^b^	μV_RMS_	5	5	5	5,0	13,2
Factor^c^		1	1	1	1	6,9
Time/configuration	s	115	115	115	113	—^d^
Total time	min	343	97	110	288	13
Average time/neuron	s	332	11	6	309	1

*AP band = 300 Hz−10 kHz. SM, switch matrix; APS, active pixel sensor. The expected number of neurons which can be mapped simultaneously in a single recording session are highlighted in boldface*.

a *Assuming one neuron per fixed electrode. Note that spike sorting should be used in dense cultures to identify single-neuron activity, which would increase this number*.

b *Total (RMS) noise consists of circuit noise, noise from dendritic Pt black electrodes (1.8 μV) (Viswam et al., 2019) as well as from background neuronal activity (4 μV). These noise sources are uncorrelated and they add up to 3.1 μV in saline (Frey et al., 2010) and to about 5 μV in the neuronal cultures cultured on our HD-MEA. From these values, we can estimate the total noise while recording from such cultures on other HD-MEA with similar electrodes*.

c *Factor by which the recording time per configuration increases to obtain a similar noise reduction after spike-triggered averaging. This factor is the square of the ratio between the total noises for each HD-MEA*.

d *Full-frame readout does not require selection configuration with different electrodes*.

e *Number of neurons was fixed at 1,000 for comparison with HD-MEAs with larger number of electrodes and switch-matrix design*.

In our culture, 53 of the 62 fixed electrodes recorded single-unit activity. However, only 28 axonal arbors could be identified, because 25 electrodes did not record enough spikes to reliably determine arbor structures (see Methods). The remaining 9 fixed electrodes recorded multi-unit activity, so that spike sorting was used to identify single-neuron activity and to obtain additional 40 axonal arbors. In total, we mapped the axonal arbors of 68 neurons. For further analysis we only used the *n* = 46 neurons with axonal arbors extending over more than 50 electrodes (**Figure 6**).

### Identification of Axonal Arbors

**Figure 3:**
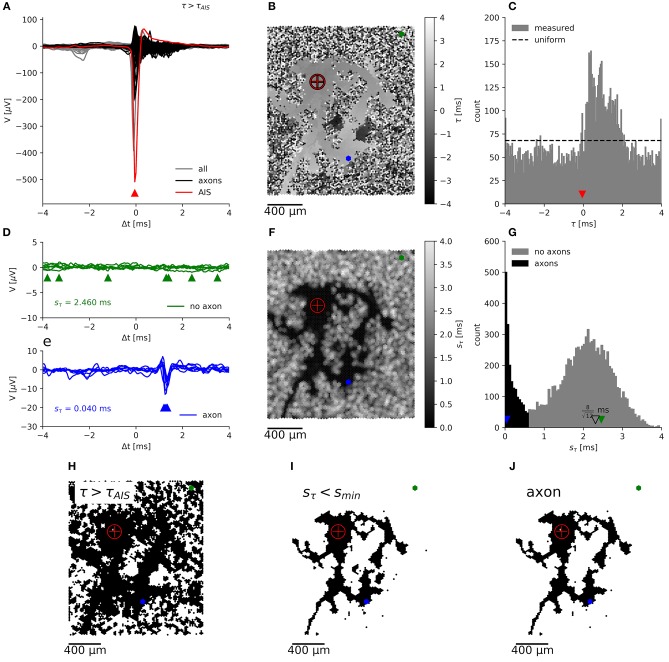
Segmentation of an axonal arbor based on the spatially correlated spontaneous activity of a single neuron. Spike-triggered averages **(A)** of signals from electrodes located close to the (proximal) AIS (red trace), close to axons (black) and for electrodes recording background activity and noise (gray). The negative peak at the AIS appears slightly earlier than at the trigger electrode. Mapping **(B)** and histogram **(C)** of the delay of the negative peak, τ, showing an irregularly shaped area with a “smooth” gray value outlining the axonal arbor, which is surrounded by a “salt-and-pepper” patterned background area. Axonal signals appear at 0 *ms* < τ < 2 *ms*. Spike-triggered averages for *N* = 7 neighboring electrodes, located in the “salt-and-pepper” region **(D)**, feature a large sample standard deviation for the delays, *s*_τ_*n*__, as compared to those located in the “smooth” region **(E)**. Mapping **(F)** and histogram **(G)** of *s*_τ_*n*__. The small irregularly shaped area outlining the axonal arbor is dark, whereas the surrounding area is displayed in lighter tones. Segmentation is done by placing the threshold *s*_*thr*_ ≈ 0.5 *ms* in the valley between the sharper peak (black), close to 0 *ms*, and the broad peak (gray) around the expected $s_{background}=8/\sqrt{12}ms$ (open triangle) for random delays. Mapping of electrodes, where the negative peak appears after the negative peak of the AIS **(H)**, with *s*_τ_*n*__ < *s*_min_ **(I)**, which record presumably axonal signals **(J)**. The crosshair symbol shows the location of the (proximal) AIS, the green and blue dots represent a patch of 7 neighboring electrodes located in the “salt-and-pepper” and “smooth” areas, respectively. Corresponding negative peaks are indicated by triangles of the same color.

**Figure 4:**
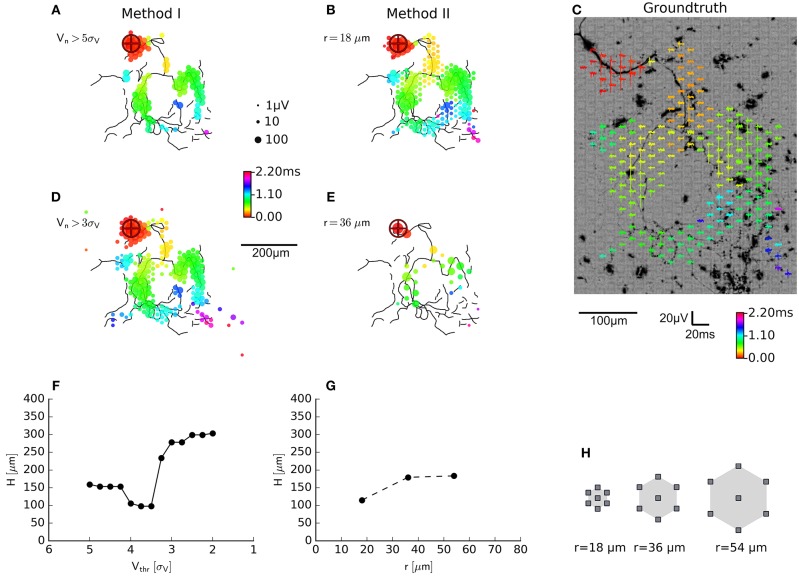
Evaluation of axon segmentation based on ground truth. Mappings and Haussdorff distance, H,are shown for method I **(A,D,F)** and II **(B,E,G)**. The high threshold, employed by method I, leads to a higher false negative rate and a larger H≈150 μm compared with H≈100 μm for method II. Lowering the threshold from 5*s*_*V*_ **(A)** to 3*s*_*V*_ **(D)** for method I leads to more false positive electrodes, far away from the axon (black outlines) and H≈ 300 μm. In contrast, method II is robust **(G)** with respect to an increased electrode distance **(H)**: increasing the distance from *r* ≈ 18 μm **(B)** to *r* ≈ 36 μm **(E)** yields a higher true positive rate and H≈ 200 μm. Axons were manually traced from fluorescence images (DsRed fluorescence displayed using an inverted grayscale) **(C)**.

Previously (see Figure 6 in Bakkum et al., 2013), axonal arbors were traced according to the occurrence of negative peaks in signal amplitudes in the spike-triggered averages of extracellularly recorded electrical signals that exceeded 5 times the background noise (*s*_*V*_). This method (hereafter termed “method I”) evaluates the spike-triggered averages for each electrode separately, without considering their spatial arrangement and signal correlations between neighboring electrodes. We used another method (hereafter termed “method II”) to benefit from the fact that the delay of the negative signal amplitude peak in the extracellularly measured potential originating from a single axon is very similar at neighboring electrodes it passes by. When we mapped the delay of these negative signal peaks in the spike-triggered averages (Figure 3A), the resulting map showed a distinct region of similar delays against a background of random delays (Figure 3B). This finding is due to the fact that signals originating from a common source, e.g., from an axon of the same neuron, are very similar across neighboring electrodes, whereas in the case of random signals the negative peak could occur anywhere in the interval [Δ*t*_*pre*_, Δ*t*_*post*_] for which the spike triggered averaging was performed (see histogram in Figure 3C). Δ*t*_*pre*_ and Δ*t*_*post*_ represent the boundaries relative to the spike triggering, so that the total spike-triggered average has the length *T* = Δ*t*_*post*_ − Δ*t*_*pre*_. To quantify the smoothness of the delay map, the delay was sampled in a neighborhood (compare Figure 4H) of hexagonal shape around each electrode, and the sample standard deviation *S*_τ_*n*__ was calculated. Note, that in the case of a uniform distribution over the interval [0, 1], the standard deviation of a sample is bounded by 0 ≤ *s* ≤ 0.5 and shows a characteristic distribution, depending on the number of observations, *N*, in each sample.

This distribution shows a sharp peak around the mean standard deviation:

$$\begin{array}{l} \bar{s_{random}}=\frac{1}{\sqrt{12}}. \end{array}$$

There is no analytical expression for this distribution, but after a coordinate transformation of the interval, it can be approximated by a beta distribution B(α,β).

In case that axonal signals are present, the delays in the neighborhood of an electrode with a negative peak at *t* are distributed in the interval $\left[t\;-\frac{r}{c};t\;+\frac{r}{c}\right]$, depending on the velocity, *c*, of the action potential and the distance, *r*, between the electrodes. Therefore, the mean of the sample standard deviation for axonal delays is:

$$\begin{array}{l} \bar{s_{axon}}=\frac{2r}{c\sqrt{12}}=\frac{r}{c\sqrt{3}}. \end{array}$$

For an HD-MEA with *r* = 18 μ*m* and a typical conduction velocity for short-range-projecting axons in the rat neocortex of 0.3−0.44 *m*/*s* (Lohmann and Rörig, 1994; Telfeian and Connors, 2003), a standard deviation of around 30 μ*s* can be expected (compare with Figure 3E). At the boundary, more and more neighboring electrodes will no more pick up the axonal signal, so that the sample standard deviation shifts toward:

$$\begin{array}{l} \bar{s_{random}}=\frac{T}{\sqrt{12}}. \end{array}$$

In our case, with *T* = 8 ms, a standard deviation of 2.3 ms for background signals would be expected (compare with Figure 3D). Empirically, the distribution of *s*_*axon*_ can be approximated by an (truncated) exponential distribution E(λ) (see below). Therefore, axons have a distribution of *s*_τ_*n*__ with a peak close to zero, which is clearly distinguishable from the distribution for the background. A threshold *s*_*min*_, placed at the local minimum in the *s*_τ_*n*__ distribution (Figure 3G), can be used to separate both populations. The electrodes with a *s*_τ_*n*__ below this threshold represent negative peaks that are consistent across neighboring electrodes (Figure 3I). If these peaks appear after the negative peak at the AIS (Figure 3H), they are assumed to originate from the axonal arbor of the neuron (Figure 3J).

For a limited number of neurons, the ground truth in the form of fluorescence images was available, so that we could compare the performance of the new method (method II; Figure 4B) with the old method (method I; Figure 4A) employing a fixed threshold at 5*s*_*V*_ (Bakkum et al., 2013). An example of a transfected neuron is shown in Figure 4C. More electrodes were selected by method II than by method I, which could be compensated for by lowering the threshold, e.g., to 3*s*_*V*_ (Figure 4D). In order to compare the electrode selection (a set of electrode coordinates, *E*) with the ground-truth axon information (a set of pixels in the image, *A*), we used the Haussdorff distance H(A,E), which is commonly used in computer vision to measure how strongly two shapes differ from each other. After registering the fluorescence image to the electrode coordinate system, we calculated

$$\begin{array}{l} H=\text{max}\left\{\underset{a\in A}{\text{sup}}\underset{e\in E}{\text{inf}}d\left(a,e\right),\underset{e\in E}{\text{sup}}\underset{a\in A}{\text{inf}}d\left(a,e\right)\right\} \end{array}$$

using the Euclidean distance *d*(*a, e*) between the coordinate $e={\left(x_{e},y_{e}\right)}^{T}$ of an electrode recording an axonal signal and the coordinate $a={\left(x_{a},y_{a}\right)}^{T}$ of the pixel representing an axon in the fluorescence image. For smaller threshold for method I lead to a larger deviation from the ground truth than method II. This was mainly due to the fact that, more electrodes far away from the axonal arbors were selected. The new method inherently relies on adjacency and rejected these “outliers” and produced more compact maps that more closely followed the ground truth. In other words, the distributions of the feature used to classify the electrical activity as either axonal signals or as background, showed a larger overlap for method I than method II. We tested the robustness of method II against the spatial distance of the electrodes by increasing the spatial extension of the neighborhood while keeping the number of electrodes in each neighborhood constant (*N* = 7). When hexagonal patterns with *r* = 2 × 18 μ*m* (Figure 4E) or *r* = 3 × 18 μ*m* distance between electrodes were selected, the distance to the ground truth only slightly increased (Figure 4G). However, it seemed that for a carefully chosen threshold (e.g., around 4.5*s*_*V*_, *H*≈100 μ*m*, Figure 4F), the original method performed as well as the new method.

**Figure 5:**
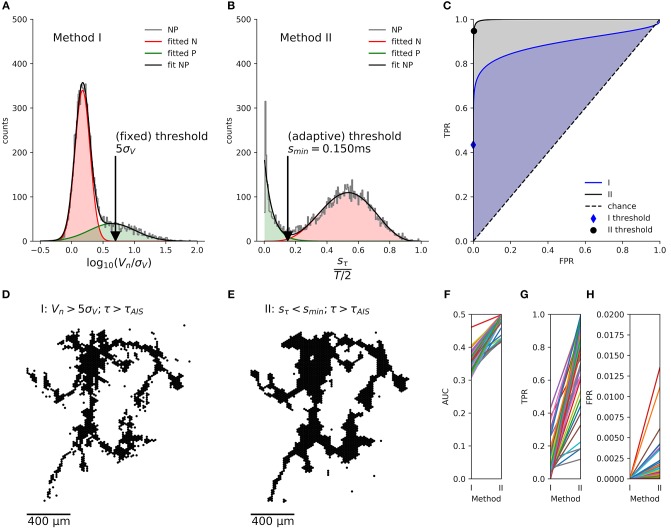
Comparison of the axon segmentation methods, based on the receiver-operator characteristics (ROC). Distributions and mappings are shown for the segmentation of an individual neuron, segmented by methods I **(A,D)** and II **(B,E)**. The empirical distribution (NP) of amplitudes *V*_*n*_ (log normalized by signal noise, σ_*V*_) and the sample standard deviations of the delays, *s*_τ_*n*__ (normalized by *T*/2) of the negative peaks were fitted (fit NP) to obtain the distributions of axonal signals (positive class, P) and background activity (negative class, N). The corresponding true-positive rate (TPR) and false-positive rate (FPR) were calculated for each possible threshold and plotted as ROC curve **(C)**. The cross depicts the position of the (fixed) threshold of method I (FPR = 0.00009, TPR = 0.7), whereas the circle indicates the (adaptive) threshold of method II (FPR = 0.011, TPR = 0.85). Method II (gray shading) performs better than method I (blue shading) as shown by the larger area under the curve (AUC). This held true for all *n* = 46 neurons **(F)**, and, although method I has a lower FPR **(H)**, its TPR **(G)** was much lower than that of method II, as it missed out on more than 50% of the axonal signals.

Due to the limits of the Haussdorff distance in estimating the quality of the extracted mappings, we also calculated the receiver-operator characteristics (ROC) (Fawcett, 2006), which is possible, as both segmentation methods are binary classifiers. We fitted the respective empirical distributions of their scores with a mixture of two partially overlapping distributions representing axonal signals (“positive” class, P) and background activity (“negative” class, N) with:

1. two normal distributions N (Figure 5A) for the segmentation with method I (Figure 5D)
  $P\left(x\right)\approx p_{N}N\left(x;\mu_{N},\sigma_{N}^{2}\right)+p_{P}N\left(x;\mu_{P},\sigma_{P}^{2}\right)\;$with $x=\log\frac{V_{n}}{s_{n}}$, μ_*N*_ < μ_*P*_
2. a beta distribution B and a truncated exponential distribution E (Figure 5B) for the segmentation with method II (Figure 5E):
  $P\left(x\right)\approx p_{N}B\left(x;\alpha_{N},\beta_{N}\right)+p_{P}E\left(x;\lambda_{P}\right)$ with $x=\frac{s_{\tau_{n}}}{T/2}$, $E\left(x;\lambda \right)=\frac{\lambda e^{-\lambda x}}{1-e^{-\lambda x}}$ for 0 ≦ *x* ≦ 1

For both methods, we used the fitted distributions to estimate the probability observing axonal signals (*P*^+^) or background activity (*P*^−^).

1. $P^{-}\left(x\right)=\;p_{N}N\left(x;\mu_{N},\sigma_{N}^{2}\right)$ and $P^{+}\left(x\right)=p_{P}N\left(x;\mu_{P},\sigma_{P}^{2}\right)\;$for method I,
2. $P^{-}\left(x\right)=p_{N}B\left(x;\alpha_{N},\beta_{N}\right)$ and $P^{+}\left(x\right)=p_{P}E\left(x;\lambda_{P}\right)$ for method II.

We then numerically calculated the cumulative distributions in order to calculate the true positive rate (*TPR*) and false positive rate (*FPR*) for each threshold, *x*, and plotted them as an ROC curve (Figure 5C). The area under the curve (*AUC*) showed that the new method consistently had a better performance than the original method for a total of *n* = 46 neurons (Figure 5F). Furthermore, the automatic threshold procedure yielded a much better *TPR* (Figure 5G) at the expense of a slightly increased false-positive rate *FPR in* comparison to the original method (Figure 5H).

## Discussion

**Figure 6:**
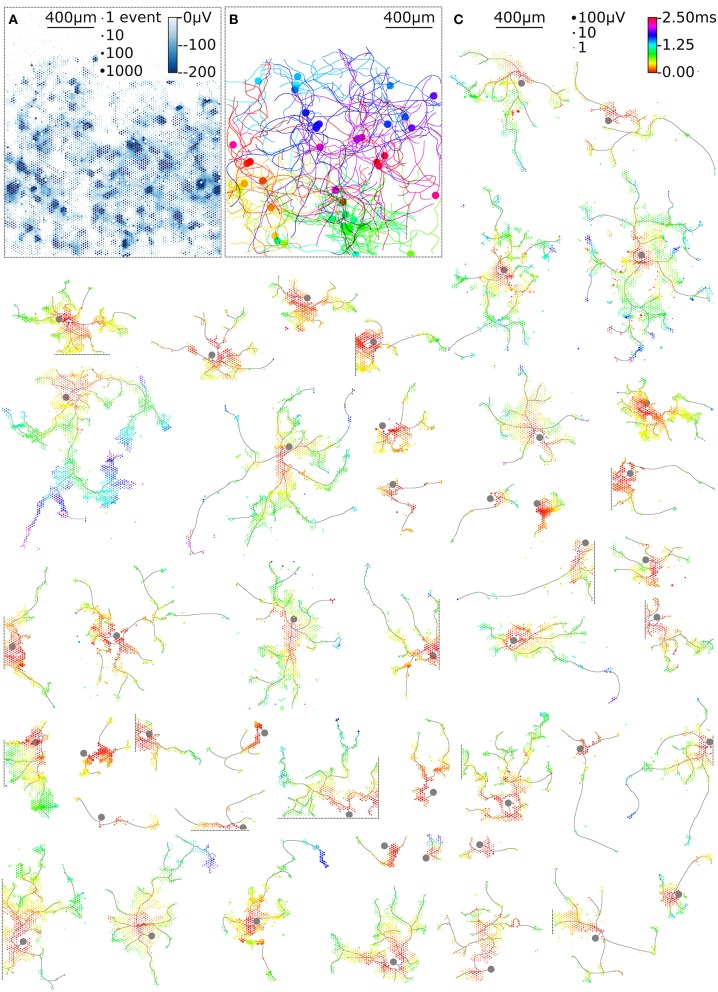
High-throughput mapping of axonal arbors. The activity map reveals the locations of the AISs of several neurons **(A)**. The circle size indicates the square-root-scaled count of spiking events per electrode. The median negative amplitude of the spikes is color-coded with a cut-off at −200 μV. Spike-triggered averaging shows the axonal footprint of 46 neurons **(C)**. Only neurons with axonal arbors extending over more than 50 electrodes are shown. The circle size indicates the square-root-scaled amplitudes of the average APs. The axonal delay is color-coded. Gray axonal contours serve as guide to the eye and have been estimated by observing the spatial movement of signal peaks in consecutive movie frames. The axonal contours of all neurons were color-coded and combined, showing the axonal arbors in the recorded neuronal network **(B)**.

HD-MEAs with more than 3,000 electrodes per square millimeter and dedicated low-noise on-chip amplifiers are suitable tools to record the electrical activity of individual axonal arbors. We first optimized a recording scheme for the switch matrix HD-MEA that relied on combinations of fixed and variable recording sites for high-throughput parallel mapping of as many neurons as possible per total recording time. The method described here shows a promising way to obtain axonal arbors at large scale from potentially all neurons during a single recording session. As an example, 68 neurons were mapped in parallel, 48 neurons of which featured large axonal arbors (Figure 6). This yield can be further improved using an HD-MEA design with an increased number of simultaneously active recording channels, decreasing the number of necessary measurement configurations and, hence, the on average the required measurement time per neuron to a few seconds (Table 1).

We then developed a method to distinguish axonal signals from the background noise. Axonal arbors reveal themselves by typical waveforms of the extracellular electric field potentials (Bakkum et al., 2013, 2014; Petersen et al., 2015; Deligkaris et al., 2016). Previously (Bakkum et al., 2013), axonal arbors were traced according to the occurrence of negative peaks in signal amplitudes in the spike-triggered averages of extracellularly recorded electrical signals that exceeded 5 times the background noise. With this threshold more than 50% of the smaller axonal signals are lost (Figure 5G). Lowering the threshold can alleviate this problem, however, the likelihood that background signals are falsely assigned to axonal arbors also increases. Some electrodes measuring spurious signals can easily be detected, if they are located at comparably large distance from electrodes that record genuine axonal signals. Typically, these false positives are removed manually after visual inspection of the recorded waveforms and their isolated location in the recording area. However, this procedure is not possible for more than a few recorded axonal arbors. Therefore, we developed a new method that compares the axonal delays on neighboring electrodes. If these delays are similar, the corresponding signals most likely originated from the same axon. If there are large differences in the delay, the corresponding signals may represent background noise. Both methods, amplitude thresholding (Bakkum et al., 2013) and the new method, worked well when we validated them by comparing their results with the morphologies of axonal arbors obtained by sparse transfection (Bakkum et al., 2013; Radivojevic et al., 2016). Upon comparing both methods according to their ROCs on a larger data set of 46 axonal arbors, the new method based on local correlations outperformed the classic amplitude thresholding. Furthermore, the new method does not require manual intervention, e.g., for setting a threshold.

The example workflow demonstrated here enables high-throughput scanning of axonal arbors and mapping of their axonal delays without the need to adjust parameters for the detection of axonal signals. This method can be extended to new generations of HD-MEAs (Ballini et al., 2014; Viswam et al., 2016; Yuan et al., 2018) and can be used to obtain data from axonal arbors of thousands of neurons within a recording session of a few hours (Table 1). Our example workflow yielded axonal arbors of 68 neurons, 46 of which featured axonal arbors extending over more than 50 electrodes (Figure 6C). These axonal arbors showed considerable variation in total length, but also in local branching patterns and axonal delays (Supplemental Figure 1). Interestingly, some neurons featured footprints in the form of disconnected patches, which could be the signature of saltatory conduction of action potentials. This observation is consistent with the reported formation of nodal components in neuronal cultures (Freeman et al., 2015), but requires further experimental validation.

Our method can be used for automated selection of neurons with suitable axonal arbors for stimulation experiments (Jäckel et al., 2017), single action potential tracking (Radivojevic et al., 2017), automated patching (Obien et al., 2019), for mapping of ion receptors by local drug application (Sasaki et al., 2011) and even for automated single-cell phenotyping of axonal conduction in human iPSC-derived neuronal cultures.

## Data Availability

The Hana (high density microelectrode array recording analysis) analysis pipeline is open source. All source code as well as example data to replicate the figures are available at: http://github.com/tbullmann/hdmea_axon. The example data consists of spike triggered-averages that were extracted from the raw recordings.

## Ethics Statement

All experimental procedures on animals were carried out in accordance with the European Council Directive of 22 September 2010 (2010/63/EU) and had been approved by the local authorities (Animal Care and Use Committee of RIKEN; QAH24-01).

## Author Contributions

TB designed the study, performed experiments, wrote the software, analyzed data, assembled figures, interpreted the results, prepared, and revised the manuscript. MR performed recording and imaging experiments. SH implemented and tested analysis algorithms. KD performed cell culture experiments. AH interpreted the results, prepared, and revised the manuscript. UF planned the study, supported the experiments, interpreted the results, and revised the manuscript.

### Conflict of Interest Statement

UF is a co-founder of MaxWell Biosystems AG, Mattenstrasse 26, Basel, Switzerland. The remaining authors declare that the research was conducted in the absence of any commercial or financial relationships that could be construed as a potential conflict of interest.

## Supplementary Material

Supplementary Figure 1 Sholl analysis for number of electrodes **(A)** and axonal delay **(B)** for *n* = 46 neurons shown in Figure 6.
