# Supplementary figures and images for: Large-Scale Mapping of Axonal Arbors Using High-Density Microelectrode Arrays
Source: Front Cell Neurosci. 2019 Sep 6;13:404. doi: 10.3389/fncel.2019.00404 (PMC6742744; doi:10.3389/fncel.2019.00404)

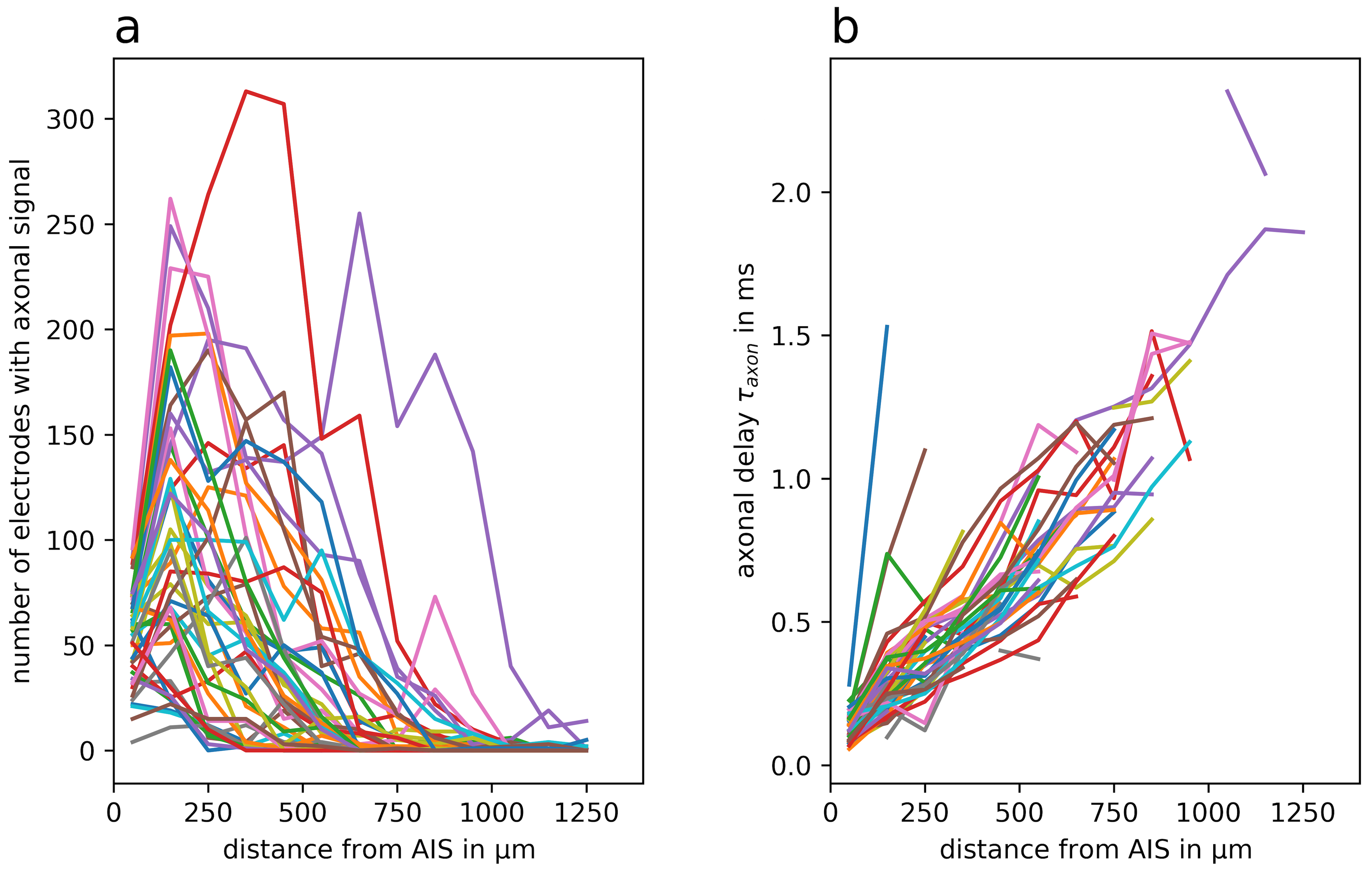

Supplement: Supplementary Figure 1 — Sholl analysis for number of electrodes (A) and axonal delay (B) for n = 46 neurons shown in Figure 6. [file Image_1.jpg]
